# Endometriosis is the independent prognostic factor for survival in Chinese patients with epithelial ovarian carcinoma

**DOI:** 10.1186/s13048-017-0363-y

**Published:** 2017-10-03

**Authors:** Tong Ren, Shu Wang, Jian Sun, Ji-Min Qu, Yang Xiang, Keng Shen, Jing He Lang

**Affiliations:** 1 Department of Obstetrics and Gynecology, Peking Union Medical College Hospital, Peking Union Medical College & Chinese Academy of Medical Sciences, Shuaifuyuan 1, Wangfujing, Dongcheng District, Beijing, 100730 People’s Republic of China; 2 Department of Pathology, Peking Union Medical College Hospital, Peking Union Medical College & Chinese Academy of Medical Sciences, Beijing, People’s Republic of China; 3 School of Public Health, Peking Union Medical College & Chinese Academy of Medical Sciences, Beijing, People’s Republic of China

**Keywords:** Endometriosis, Epithelial ovarian carcinoma, Prognostic factor

## Abstract

**Background:**

Clinico-pathological characteristics and possible prognostic factors among women with epithelial ovarian carcinoma (EOC) with or without concurrent endometriosis were explored.

**Method:**

We retrospectively identified 304 patients with EOC treated primarily at Peking Union Medical College Hospital with median follow-up time of 60 months.

**Results:**

Of 304 patients with EOC, concurrent endometriosis was identified in 69 (22.7%). The patients with concurrent endometriosis were younger and more probably post-menopausal at onset, were less likely to have abdominal distension, with significantly lower level of pre-surgery serum Ca125 and less possibility of having the history of tubal ligation. The women with concurrent endometriosis group were more likely to have early stage tumors (88.41% versus 52.77%), receive optimal cytoreductive surgery (92.75% versus 71.06%), and less likely to have lymph node metastasis or to develop platinum resistance disease (7.25% versus 14.89%, and 7.35% versus 20%), when compared with women without coexisting endometriosis. The univariate analysis showed that concurrent endometriosis was a prognostic factor for overall survival (OS) and disease-free survival (DFS), but this association just remained in the DFS by multivariate analysis. Besides, multivariate analysis also showed that FIGO stage, residual disease, chemotherapy cycles, chemotherapy resistance and concomitant hypertension were the independent impact factors of OS for EOC patients; whereas FIGO stage, lymphadenectomy, residual disease, coexisting endometriosis and chemoresistance were independent impact factors of DFS for those patients.

**Conclusions:**

EOC patients with concurrent endometriosis showed distinct characteristics and had longer overall survival and disease-free survival when compared with those without endometriosis. Endometriosis was the independent prognostic factor for DFS for patients in this series.

## Background

Endometriosis (EM) is one of the most common benign gynecological diseases, with an incidence as high as 10–15% in women of childbearing age. EM typically manifests as masses, pain, and infertility in clinical practice, seriously affecting physical and psychological health as well as the quality of life of women of childbearing age. Nonetheless, attention has long been paid to the malignant potential of EM [1, 2]. In recent years, the association between malignant transformation of EM and the development of particular types of epithelial ovarian cancer (EOC) has become a hot topic in basic clinical research [3–6]. EM may represent a precancerous lesion of ovarian clear cell carcinoma (OCCC) or ovarian endometrioid carcinoma (OEC). As a potential “precancerous state”, EM is closely associated with OCCC and OEC [7–9]. Endometriosis-associated ovarian carcinoma(EAOC) may be a special type of OC that shows different clinicopathological and prognostic features compared with ordinary OC [10, 11]. The majority of existing clinical case studies have involved a small sample size and short follow-up time. In the present study, we compared the clinicopathological features and prognosis of OCCC and OEC with and without EM, using data from patients undergoing primary treatment for epithelial OC in our hospital.

## Methods

By reviewing the medical charts, we retrospectively identified 186 patients with OEC and 118 patients with OCCC who were primarily treated and received surgery at the Division of Gynecological Oncology of the Department of Obstetrics and Gynecology at Peking Union Medical College Hospital between January 2000 and January 2012. All patients received surgery and chemotherapy and were followed up at our institution. The patient follow-up period ended in March 2017. This study was approved by the University Institutional Review Board.

Here, we defined EOC with concurrent endometriosis as the presence of ovarian cancer and endometriosis identified histologically in the same ovary, the presence of endometriosis in one ovary and of ovarian cancer in the contralateral ovary, or the presence of ovarian cancer and extraovarian pelvic endometriosis (eg, peritoneal endometriosis). According to the pathological criteria listed above, we identified 68 of patients with ovarian carcinoma with concurrent EM (EAOC group), The remaining 236 patients had no pathological evidence of endometriosis (non-EAOC group).

The collected clinico-pathological data were compared between two groups as shown in Table 1. The 304 patients were subjected to surgical-pathologic restaging based on the International Federation of Gynecology and Obstetrics (FIGO) staging guidelines for ovarian cancer (2013) [12]. For statistical analysis, FIGO stage categories were classified into early stage (FIGO stages I and II subjects) and late stage (FIGO stages III to IV).

**Table 1:** Clinical and morphological characteristics of EOC patients with or without EM

	NON-EAOC	EAOC	*P*
Numbers	236	68	
Age(year)(median[IQR])	52.0 [44.00,62.00]	45.00 [40.00,49.50]	<0.001^a^
Menopause			<.0001^a^
Pre-	108 (45.96%)	53 (76.81%)	
Post-	127 (54.04%)	16 (23.19%)	
Gravid			0.6506
0	26 (11.06%)	9 (13.04%)	
> =1	209 (88.94%)	60 (86.96%)	
Parity			0.5637
0	41 (17.45%)	10 (14.49%)	
> =1	194 (82.55%)	59 (85.51%)	
Symptoms			
Abdominal pain			0.3989
Yes	76 (32.34%)	17 (24.64%)	
No	158 (67.23%)	52 (75.36%)	
Bloating			0.0193^a^
Yes	54 (22.98%)	7 (10.14%)	
No	181 (77.02%)	62 (89.86%)	
Palpable mass			0.2629
Yes	59 (25.11%)	22 (31.88%)	
No	176 (74.89%)	47 (68.12%)	
Incidental finding			0.1232
Yes	33 (14.04%)	15 (21.74%)	
No	202 (85.96%)	54 (78.26%)	
Irregular menstruation			0.1111
Yes	19 (8.09%)	10 (14.49%)	
No	216 (91.91%)	59 (85.51%)	
Postmenopausal bleeding			0.0610
Yes	18 (7.66%)	1 (1.45%)	
No	217 (92.34%)	68 (98.55%)	
Cachexia			0.0814
Yes	10 (4.26%)	0 (0%)	
No	225 (95.74%)	69 (100%)	
Abnormal vaginal discharge			0.4420
Yes	2 (0.85%)	0 (0%)	
No	233 (99.15%)	69 (100%)	
Pre-surgery Ca125 (IU/ml) (median[IQR])	256.20 [62.96, 959.30]	89.50 [37.80, 346.85]	<0.001^a^
Ca125 in normal range			0.2110
Yes	39 (16.6%)	16 (23.19%)	
No	196 (83.4%)	53 (76.81%)	
Tumor size (cm) (median[IQR])	9.00 [6.00,13.00]	10.00 [7.00,15.000]	0.245
Side of ovarian tumor			0.3904
Unilateral	161 (68.51%)	51 (73.91%)	
Bilateral	74 (31.49%)	18 (26.09%)	
Breast cancer history			0.1204
Yes	8 (3.4%)	0 (0%)	
No	227 (96.6%)	69 (100%)	
HT history			0.2433
Yes	45 (19.15%)	9 (13.04%)	
No	190 (80.85%)	60 (86.96%)	
DM history			0.2263
Yes	16 (6.81%)	2 (2.9%)	
No	219 (93.19%)	67 (97.1%)	
Hysterectomy history			0.1469
Yes	7 (2.98%)	0 (0%)	
No	228 (97.02%)	69 (100%)	
Tube ligation history			0.0379^a^
Yes	14 (5.96%)	0 (0%)	
No	221 (94.04%)	69 (100%)	

Abbreviation: *EOC*, epithelial ovarian carcinoma; *EM*, endometriosis; *EAOC*, endometriosis-associated ovarian cancer; *IQR*, InterQuartile Range; *Ca125*, cancer antigen 125; *HT*, hypertension; *DM*, diabetic mellitus;

^a^the difference reached statistical significance

Besides, synchronous tumors of the ovary and endometrium were found and analyzed in this series. The criteria of Young and Scully [13] was used for interpretation of synchronous primary tumors of both organs or of metastasis from one organ to the other. When the pathologic study reveals similar types, the differentiation between the 2 separate primary cancers or 1 single advanced cancer with metastasis is much more difficult. Herein, we apply standardized criteria to differentiate the 2 synchronous cancers, rather than 1 cancer metastases [(1) both tumors are confined to primary sites; (2) no direct extension between the tumors; (3) no lymphovascular tumor emboli; (4) no or only superficial myometrial invasion of the endometrial lesion; and (5) no distant metastasis] [14, 15].

Disease-free survival (DFS) was defined as the time interval from the date of primary surgery to the date of disease progression and/or recurrence. Overall survival (OS) was defined in months as the date of the primary surgery to the date of death or censoring at the date of last contact.

Continuous variables were analyzed using Mann–Whitney U test. Categorical variables were analyzed by using test or Fisher’s exact test. Receiver Operating Characteristic (ROC) curve was constructed to define the optimal cutoff value for stratifying and grouping continuous variables. Survival comparisons were obtained using the log-rank test in an unadjusted Kaplan-Meier model. Cox proportional hazards regression was used for multivariate analysis. Variables included in this analysis were those found to be statistically significant in the univariate analyses. Hazards ratios and 95% confidence intervals (CIs) were used to calculate the relative risk of death or relapse for each variable of interest while adjusting for other covariates. All *P* values reported were 2 tailed, and a *P* value of 0.05 or less was considered as statistical significance.

## Results

Table 1 shows the statistical results for the clinical information from the two groups of patients. The patients in EAOC group exhibited an median age of onset of 45 years, which was 7 years younger than that of the patients in non-EAOC group; this between-group difference was statistically significant (*P* < 0.001). Compared with the EAOC patients, a higher proportion of non-EAOC patients were post-menopausal at onset (*P* < 0.001). No significant difference in the number of pregnancies was found between the two groups. The leading three most common referral symptoms for women in EAOC group were palpable mass(31.88%), abdominal pain(24.64%) and incidental finding(21.74%), while which for ones in Non-EAOC group were respectively abdominal pain(32.34%), palpable mass (25.11%), bloating (22.98%). Bloating was the symptom in a greater proportion of patients in non-EAOC group compared with ones in EAOC group (*P* = 0.0193), whereas no significant differences in other symptoms were found between the two groups.

The patients of EAOC group showed significantly lower preoperative CA125 levels than non-EAOC group (median[IQR], 89.50[37.80, 346.85]mIU/ml vs 256.20[62.96, 959.30] mIU/ml, *P* < 0.001). But the proportion of patients presented CA125 levels within the normal range was not significantly different (23.19% vs. 16.6%, *P* = 0.2110). No significant differences were found between the two groups in terms of tumor size or tumor side, and neither difference seen in the medical history or complication of breast cancer, hypertension (HT), diabetic mellitus (DM) and previous surgery of hysterectomy. However, there was a significant difference between the two groups in terms of tubal ligation (non-EAOC vs EAOC, 5.96% vs 0%, *P* = 0.0379).

Table 2 summarizes the surgico-pathological data. In EAOC group, 71.01% of patients were at FIGO stage I, while 88.41% had early (stage I + II) cancer. In non-EAOC group, 47.23% of the patients displayed advanced cancer. The between-group difference in tumor stage reached statistical significance (*P* < 0.001). The rate of performing lymphadenectomy in EAOC group was significantly higher than in non-EAOC group (92.75% vs 80.43%, *P* = 0.0160). Among the 68 patients in EAOC group, OCCC accounted for 53.62%, OEC for 46.38%; the non-EAOC patients mainly had OEC; these two types of cancer accounted for 34.47% and 65.53% of the patients, respectively (*P* = 0.0041). Besides, the rate of having concurrent endometrial disorders between two groups was similar (*P* = 0.0808), but the rate of concurrent endometrial carcinoma in non-EAOC group was higher than EAOC group (23.8% vs 7.4%, *P* = 0.051).

**Table 2:** The surgico-pathological characters and treatment-related variables of EOC patients with or without EM

	NON-EAOC	EAOC	*P*
FIGO Stage^a^			<.0001*
I	92 (39.15%)	49 (71.01%)	
II	32 (13.62%)	12 (17.39%)	
III	97 (41.28%)	7 (10.14%)	
IV	14 (5.96%)	1 (1.45%)	
Early or late Stage			<.0001*
I + II	124 (52.77%)	61 (88.41%)	
III + IV	111 (47.23%)	8 (11.59%)	
Lymphadenectomy			0.0160*
Yes	189 (80.43%)	64 (92.75%)	
No	46 (19.57%)	5 (7.25%)	
Residual disease			0.0002*
No or <1 cm	167 (71.06%)	64 (92.75%)	
> 1 cm	68 (28.94%)	5 (7.25%)	
Metastasis of lymph node			0.0060*
Yes	35 (14.89%)	5 (7.25%)	
No	154 (65.53%)	59 (85.51%)	
Not-dissected	46 (19.57%)	5 (7.25%)	
Histotype			0.0041*
Clear cell	81 (34.47%)	37 (53.62%)	
Endometrioid	154 (65.53%)	32 (46.38%)	
Endometrial disorders			0.0808*
Yes	45 (19.15%)	7 (10.14%)	
No	190 (80.85%)	62 (89.86%)	
Endometrial cancer			0.051
Yes	25 (23.8%)	2 (7.4%)	
No	211 (76.2%)	66 (92.6%)	
Chemotherapy			0.6250
Platinum-based	224 (95.32%)	67 (97.1%)	
Other regimen	3 (1.28%)	0 (0%)	
No chemotherapy	8 (3.4%)	2 (2.9%)	
Chemo Cycles	6.36 ± 2.33	5.70 ± 2.05	0.039*
Chemo-resistance			0.0149*
Yes	47 (20%)	5 (7.35%)	
No	188 (80%)	63 (92.65%)	

Abbreviation: *EOC*, epithelial ovarian carcinoma; *FIGO*, International Federation of Gynecology and Obstetrics; *EM*, endometriosis; *EAOC*, endometriosis-associated ovarian cancer

*the difference reached statistical significance

^a^according to the classification system of FIGO staging (2013 version)

The proportion of patients who received postoperative chemotherapy and platinum-based chemotherapy between two groups were likely. The mean cycles(±SD) of platinum-based chemotherapy were significantly divergent between two groups (6.36 ± 2.33 in non-EAOC vs 5.70 ± 2.05 in EAOC, *P* = 0.039). We defined chemo-resistance as tumor recurrence or progression within 6 months after the last chemotherapy. In this study, the rate of occurring chemo-resistance in non-EAOC group was significantly higher than in non-EAOC group (20% vs 7.35%, *P* = 0.0149).

With an median(IQR) follow-up of 60[29,90] months, the entire study cohort observed 23.03% of deaths due to disease, and 47.37% of patients developed relapse disease with the median(IQR) time of DFS of 39[13,76] months.

**Fig. 1:**
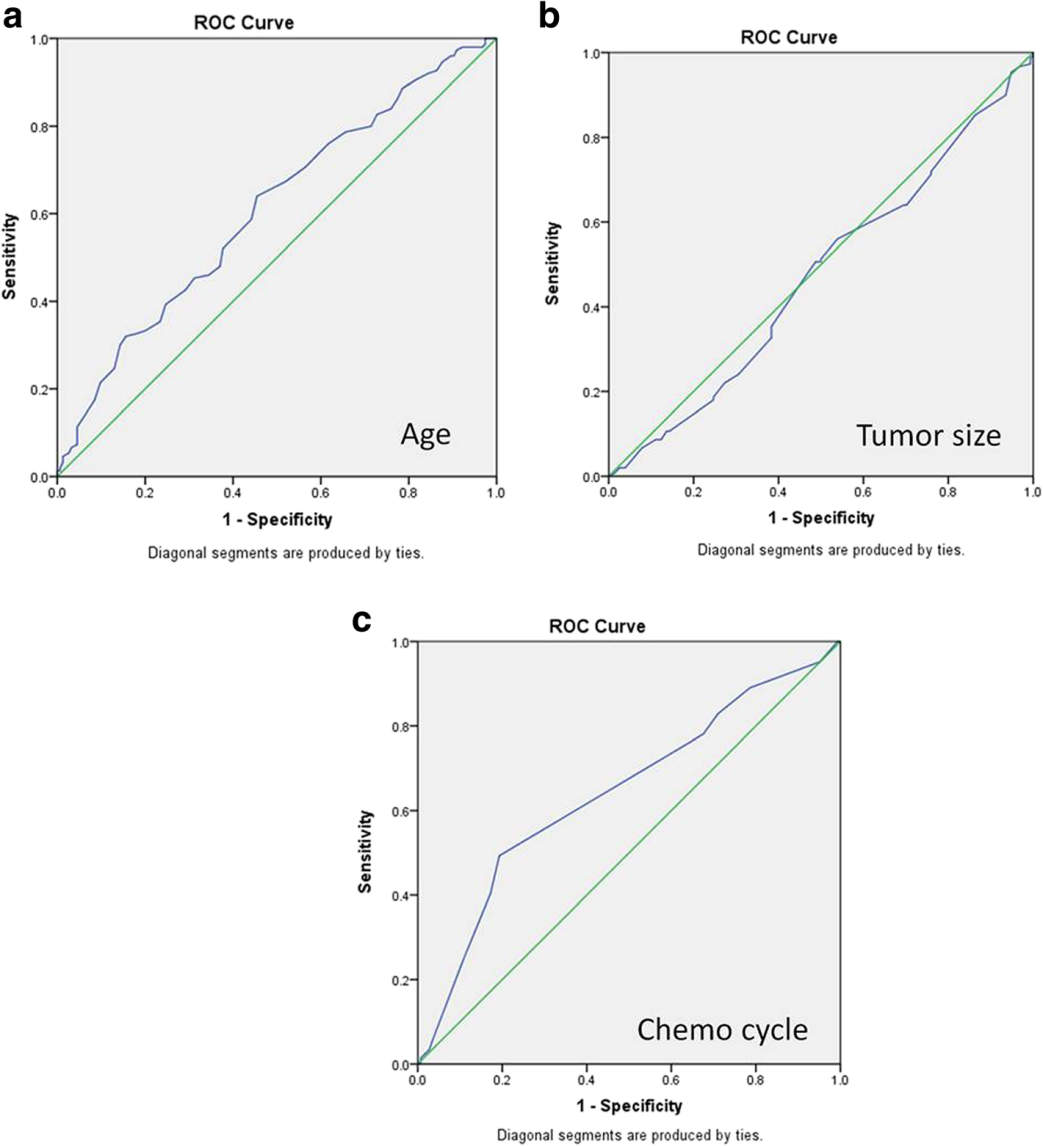
**a** ROC curve for age of disease onset in DFS; **b**. ROC curve for tumor size in DFS; **c**. ROC curse for cycles of platinum-based chemotherapy in DFS

Receiver Operating Characteristic (ROC) curve constructed to stratify the continuous variable including the age of onset, tumor size, and the courses of platinum regimen received. As a result, the optimal cutoff value was defined as 49 years for age, 10 cm for tumor size, 3 for chemotherapy courses (Showed in Fig. 1).

**Fig. 2:**
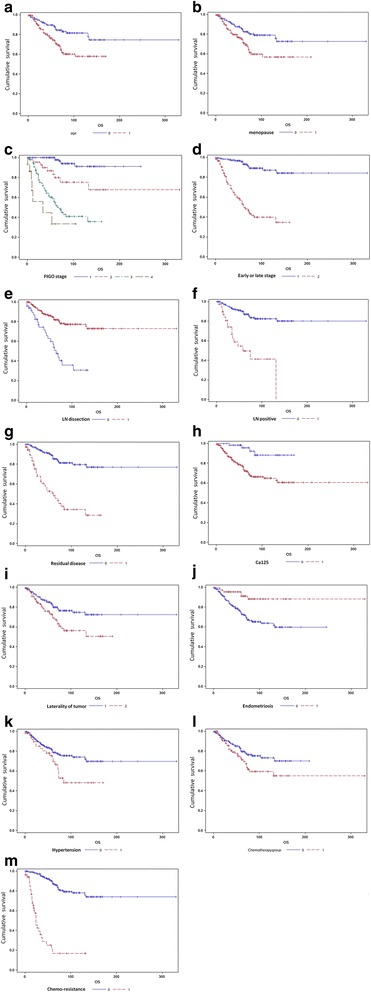
A univariate analysis of overall survival in patients with EOC. Kaplan-Meier survival curves showing the effects of age (**a**), menopausal status (**b**), FIGO stage (**c**), early or late stage (**d**), lymphadenectomy (**e**), metastasis of LN (**f**), residual disease (**g**), normal level of Ca125(**h**), unilateral or bilateral tumor (**i**), endometriosis (**j**), hypertension (**k**), chemotherapy courses (**l**) and chemo-resistance (**m**) for OS

**Fig. 3:**
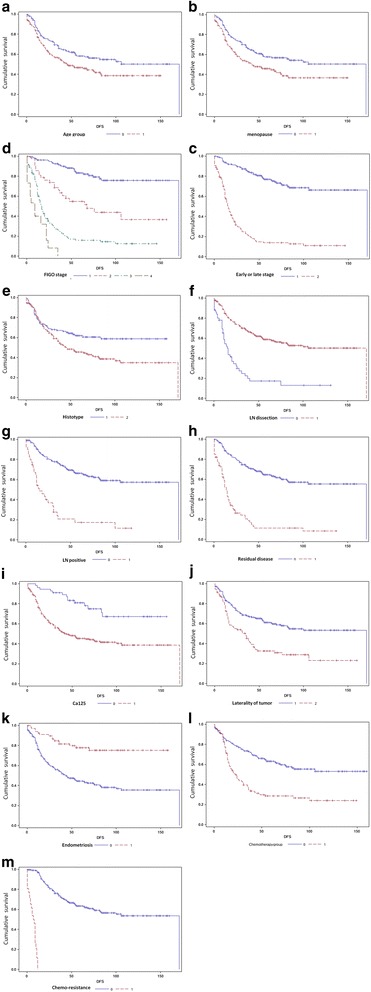
A univariate analysis of disease-free survival in patients with ovarian clear cell and endometrioid carcinoma. Kaplan-Meier survival curves showing the effects of age (**a**), menopausal status (**b**), FIGO stage (**c**), early or late stage (**d**), histotype (**e**), lymphadenectomy (**f**), metastasis of LN (**g**), residual disease (**h**), Ca125 in normal range (**i**), unilateral or bilateral tumor (**j**), endometriosis (**k**), chemotherapy courses (**l**) and chemo-resistance (**m**) for DFS

As seen in Tables 3 and 4, the univariate analysis showed that concurrent endometriosis was a prognostic factor for overall survival (OS, *P* = 0.0010) and disease-free survival (DFS, *P* < 0.0001). Besides, the following variables were showed relating to either OS or PFS, including age (*P* = 0.0013, *P* = 0.0194), menopausal status (*P* = 0.0043, *P* = 0.0052), FIGO stage (*P* < 0.0001, *P* < 0.0001), early or late stage (*P* < 0.0001, *P* < 0.0001), lymphadenectomy (*P* < 0.0001, *P* < 0.0001), metastasis of LN (*P* < 0.0001, *P* < 0.0001), residual disease (*P* < 0.0001, *P* < 0.0001), normal level of Ca125 (*P* = 0.0009, *P* < 0.0001), unilateral or bilateral tumor (*P* = 0.0063, *P* < 0.0001), chemotherapy courses (*P* = 0.0373, *P* < 0.0001) and chemo-resistance (*P* < 0.0001, *P* < 0.0001) (showed in Fig. 2). And the correlation was also seeen in OS and hypertension, but not for PFS (showed in Fig. 3).

**Table 3:** Univariate analysis of overall survival among EOC patients

Variable	Category	N(%)	OS (Median[IQR])	Survival rate(%)	*P*
Age y	<49	138 (45.39)	69.5 [38,102]	84.06	0.0013*
	> = 49	166 (54.61)	53 [23,78]	71.08	
Menopausal status	Pre	161 (52.96)	68 [38,102]	81.99	0.0043*
	Post	143 (47.04)	53 [22,77]	71.33	
Gravidity	0	35 (11.51)	60 [34,107]	82.86	0.3070
	> = 1	269 (88.49)	60 [27,89]	75.84	
Parity	0	51 (16.78)	70 [40,96]	84.31	0.1016
	> = 1	253 (83.22)	58 [26,86]	75.1	
Stage	Early	185 (60.86)	69 [47,102]	91.35	<.0001*
	Late	119 (39.14)	38 [18,72]	53.78	
FIGO Stage	I	141 (46.38)	72 [51,102]	95.74	<.0001*
	II	44 (14.47)	60 [33,110]	79.55	
	III	104 (34.21)	41.5 [20,73.5]	53.85	
	IV	15 (4.93)	10 [5,53]	53.33	
Histotype	Clear cell	118 (38.82)	58.5 [27,88]	78.81	0.6479
	Endometrioid	186 (61.18)	60.5 [31,92]	75.81	
Lymphadenectomy	Yes	51 (16.78)	38 [12,69]	52.94	<.0001*
	No	253 (83.22)	62 [34,91]	81.82	
Metastasis of LN	Positive	40 (13.16)	32.5 [15,59]	57.5	<.0001*
	Negative	213 (70.07)	68 [44,101]	86.38	
Residual disease	>1 cm	73 (24.01)	33 [15,65]	50.68	<.0001*
	None or <1 cm	231 (75.99)	66 [38,98]	85.28	
CA125 normal	Yes	55 (18.09)	73 [52,102]	92.73	0.0009*
	No	249 (81.91)	58 [24,87]	73.49	
Tumor size(cm)	<10	153 (50.33)	60 [32,90]	77.12	0.8933
	≥10	151 (49.67)	60 [26,88]	76.82	
Tumor side	Unilateral	212 (69.74)	61 [33.5,96.5]	80.66	0.0063*
	Bilateral	92 (30.26)	53 [24,84.5]	67.39	
Endometriosis	No	235 (77.30)	57 [24,88]	72.77	0.0010*
	Yes	69 (22.70)	67 [42,101]	91.3	
Endometrial disorders	No	252 (82.89)	60 [29.5,91]	75.0	0.2184
	Yes	52 (17.11)	60 [29,85.5]	84.62	
Endometrial cancer	No	252 (82.89)	60 [26.5,91]	74.6	0.2321
	Yes	27 (8.88)	60 [40,96]	85.19	
Hypertension	No	250 (82.24)	60 [32,92]	79.6	0.0132*
	Yes	54 (17.76)	57.5 [19,81]	64.81	
Chemotherapy cycle	<4	191 (62.83)	61 [34,91]	80.1	0.0373*
	≥4	100 (32.89)	53 [23.5,85]	70	
Chemo-resistance	No	252 (82.89)	67 [44,99.5]	84.52	<.0001*
	Yes	52 (17.11)	19 [11,28.5]	40.38	

*P* values were cultivated by Kaplan-Meier analysis

**Table 4:** Univariate analysis of disease-free survival among patients

Variable	Category	N(%)	OS (Median(IQR))	Survival rate(%)	*P*
Age y	<49	141 (46.38)	52 [16,87]	57.45	0.0194^a^
	> = 49	163 (53.62)	31 [12,65]	47.85	
Menopausal status	Pre	161 (52.96)	50 [17,87]	57.14	0.0052^a^
	Post	143 (47.04)	26 [11,64]	46.85	
Gravidity	0	35 (11.51)	44 [16,90]	51.43	0.6329
	> = 1	269 (88.49)	39 [12,75]	52.79	
Parity	0	51 (16.78)	51 [15,86]	52.94	0.5262
	> = 1	253 (83.22)	38 [12,75]	52.57	
Stage	Early	185 (60.86)	59 [36,88]	74.59	<.0001^a^
	Late	119 (39.14)	14 [8,28]	18.49	
FIGO Stage	I	141 (46.38)	62 [44,90]	81.56	<.0001^a^
	II	44 (14.47)	42 [17.5,80]	50	
	III	104 (34.21)	15 [9,30.5]	20.19	
	IV	15 (4.93)	9 [0.5,22]	6.67	
Histotype	Clear cell	118 (38.82)	52.5 [14,85]	61.86	0.0091^a^
	Endometrioid	186 (61.18)	34.5 [12,70]	46.24	
Lymphadenectomy	Yes	51 (16.77)	12 [6,26]	23.53	<.0001^a^
	No	253 (83.22)	48 [17,82]	58.1	
Metastasis of LN	Positive	213 (70.07)	54 [24,86]	64.79	<.0001^a^
	Negative	40 (13.16)	13 [7,31]	22.5	
Residual disease	>1 cm	231 (75.99)	53 [22,84]	17.81	<.0001^a^
	None or <1 cm	73 (24.01)	12 [5,23]	63.2	
CA125 normal	Yes	55 (18.09)	64 [48,97]	74.55	<.0001^a^
	No	249 (81.91)	30 [12,70]	47.39	
Tumor size(cm)	<10	153 (50.33)	40 [13,77]	50.33	0.7834
	≥10	151 (49.67)	38 [13,75]	54.3	
Tumor side	Unilateral	212 (69.74)	50.5 [14.5,83]	60.85	<.0001^a^
	Bilateral	92 (30.26)	27.5 [12,55.5]	33.7	
Endometriosis	No	235 (77.30)	31 [12,69]	44.68	<.0001^a^
	Yes	69 (22.70)	60 [36,89]	78.26	
Endometrial disorders	No	252 (82.89)	38 [13,75.5]	51.19	0.3357
	Yes	52 (17.11)	48 [13,76]	57.69	
Endometrial cancer	No	252 (82.89)	38 [13,75.5]	51.19	0.2503
	Yes	27 (8.88)	49 [18,86]	59.26	
Hypertension	No	250 (82.24)	41 [14,76]	52.8	0.4164
	Yes	54 (17.763)	29.5 [11,68]	50	
Chemotherapy cycle	<4	191 (62.83)	51 [19,83]	61.78	<.0001^a^
	≥4	100 (32.89)	19 [11,45]	32	
Chemotherapy resistence	No	252 (82.89)	51.5 [24,84]	63.1	<.0001^a^
	Yes	52 (17.11)	7.5[3,10]	0	

Abbreviation: *EOC*, epithelial ovarian carcinoma; *FIGO*, International Federation of Gynecology and Obstetrics

^a^the difference reached statistical significance. *P* values were cultivated by Kaplan-Meier analysis

Multivariate analysis showed coexisting endometriosis was the independent risk factor of DFS for this series of patients (*P* = 0.0233), but not OS (*P* = 0.4038). And the Tables 5 and 6 also revealed that FIGO stage (*P* = 0.0001), chemotherapy cycles (*P* = 0.0058), chemotherapy resistance (*P* < 0.0001) and concomitant hypertension (*P* = 0.0102) were the independent impact factors of OS, whereas FIGO stage (*P* < 0.0001), lymphadenectomy (*P* = 0.0084), residual disease (*P* = 0.0165), and chemoresistance (*P* < 0.0001) were independent impact factors of DFS for EOC patients in this study.

**Table 5:** Multivariable analysis of overall survival by Cox regression model for EOC patients

Variable	β	Standard Error	Chi-Square	*P*	HR(95% CI)
Age group	−0.72	0.43	2.83	0.0923	0.49(0.21 ~ 1.13)
Menopause	0.76	0.44	3.00	0.0835	2.14(0.9 ~ 5.06)
Stage (early or late)	1.48	0.39	14.63	0.0001*	4.40(2.06 ~ 9.4)
Histotype	−0.36	0.28	1.69	0.1941	0.7(0.4 ~ 1.2)
Lymph node positive	0.11	0.38	0.09	0.7667	1.12(0.53 ~ 2.36)
Lymphadenectomy	0.43	0.33	1.73	0.1887	1.54(0.81 ~ 2.95)
Residual disease	0.51	0.28	3.47	0.0625	1.67(0.97 ~ 2.87)
CA125 normal	0.68	0.56	1.51	0.219	1.98(0.67 ~ 5.89)
Tumor side	0.29	0.27	1.17	0.2797	1.34(0.79 ~ 2.28)
Endometriosis	−0.39	0.47	0.70	0.4038	0.68(0.27 ~ 1.69)
Chemotherapy course	−0.78	0.28	7.61	0.0058*	0.46(0.26 ~ 0.8)
Chemoresistance	1.94	0.32	37.68	<.0001*	6.96(3.75 ~ 12.93)
Hypertension	0.77	0.30	6.61	0.0102*	2.16(1.2 ~ 3.89)

*the difference reached statistical significance. *P* values were cultivated by Cox regression analysis. The overall test of the above model showed the model was significance, *p* < 0.0001

**Table 6:** Multivariable analysis of disease-free survival by Cox regression model for EOC patients

Variable	β	Standard Error	Chi-Square	*P*	HR(95% CI)
Age group	0.13	0.30	0.19	0.6653	1.14(0.63 ~ 2.07)
Menopause	0.14	0.29	0.22	0.6377	1.15(0.65 ~ 2.03)
Stage (early or late)	1.03	0.26	15.78	<.0001*	2.79(1.68 ~ 4.64)
Histotype	0.26	0.19	1.87	0.1713	1.3(0.89 ~ 1.9)
Lymph node positive	0.22	0.24	0.84	0.3605	1.25(0.77 ~ 2.02)
Lymphadenectomy	0.61	0.23	6.96	0.0084*	1.84(1.17 ~ 2.89)
Residual disease	0.48	0.20	5.75	0.0165*	1.62(1.09 ~ 2.39)
CA125 normal	0.25	0.31	0.68	0.4086	1.29(0.71 ~ 2.36)
Tumor side	0.26	0.19	1.96	0.1619	1.3(0.9 ~ 1.87)
Endometriosis	−0.66	0.29	5.15	0.0233*	0.52(0.29 ~ 0.91)
Chemotherapy group	−0.03	0.19	0.02	0.8773	0.97(0.66 ~ 1.42)
Chemoresistance	3.77	0.42	81.43	<.0001*	43.3(19.1 ~ 98.15)

*the difference reached statistical significance. *P* values were cultivated by Cox regression analysis. The overall test of the above model showed the model was significance, *p* < 0.0001

## Discussion

In this study, 68 of 304 patients enrolled exhibited an association with EM, which accounted for 22.37% of the patients with EOC. The incidence of EAOC in our patients was close to the values reported by Nezhat [16].

With respect to clinical features, we found that the patients in EAOC group exhibited a markedly lower median age of onset than the non-EAOC patients (45 years vs. 52 years); most EAOC patients were premenopausal at onset (76.81% vs. 45.96%). This is in agreement with the results of Garrett [17], Lim [18], and Scarfone [19]. We wonder if this between-group difference may be due to the following factors: the EAOC patients presented more evident clinical symptoms and tended to undergo pelvic examination more frequently, indirectly moving the timing of the detection of ovarian tumors forward. However, in the present study, we found no significant difference in clinical symptoms between the two groups, except that a relatively high proportion of non-EAOC patients exhibited bloating as the first symptom (22.98% vs. 10.14%). From this aspect, We tend to believe that EAOC shows a younger age of onset due to intrinsic mechanisms, rather than simply early detection [9, 10].

Median preoperative serum CA125 levels were significantly different between the two groups of patients (256.20 vs. 89.50 U/ml), a finding that is inconsistent with the results of Scarfone [19] and Mangili [20]. It is currently thought that CA125, a predictor of malignant ovarian tumors, is divergent among the different histological type of tumors [21, 22]. Therefore, precise classification of epithelial OC, followed by the identification of new tumor markers, will have important clinical value, and further relevant research is expected to be conducted.

Comparison of surgical histories between groups revealed that a markedly higher proportion of non-EAOC patients relative to EAOC patients underwent tubal ligation, but no difference for hysterectomy. Van Gorp [23], Nagle [24], and Rice [25, 26] have shown that hysterectomy or tubal ligation can decrease the incidence of EM and EAOC to varying degrees, which may explain our results. That is, tubal ligation may have reduced the development of EAOC in this series of patients. This result is interesting and remains to be verified in a large-sample, prospective, long-term study.

Similar to the results of numerous studies [18–20], we found that most OCCC and OEC patients of the EAOC group (71.01%) had FIGO I stage cancer at diagnosis. A markedly higher proportion of patients had early (stage I–II) cancer in the EAOC group compared with the non-EAOC group (88.41% vs. 52.77%). It is therefore easier to perform lymphadenectomy and to achieve satisfying debulking surgery in the former group than in the latter group (respectively 92.75% vs 80.43% and 92.75% vs 71.06%). As discussed previously, it is unclear whether the earlier clinicopathological stage of EAOC patients is due to early detection or the biological behavior of the tumor. Currently, there is no evidence showing that this patient population exhibits particular first symptoms or a specific medical history leading to early detection of tumors. Therefore, we tend to believe that EAOC may possess certain unique cellular and molecular biological characteristics that result in slow progression of the disease [10].

Although the cases enrolled in this study spanned a period of more than 12 years, during which there was no major revision in the chemotherapy regimens and strategies for treating OC. We evaluated the situation of postoperative chemotherapy regimens patients had received. Most patients underwent postoperative platinum-based chemotherapy in the two groups (92.9% vs. 95.8%). The results showed no significant difference between the two groups with regard to receiving postoperative adjuvant chemotherapy. Nevertheless, EAOC patients received less courses of chemotherapy(mean ± SD, 5.70 ± 2.05 vs 6.36 ± 2.33 cycles) and a markedly lower percentage of EAOC patients received were platinum resistant, when compared with non-EAOC patients (7.35% versus 20%). The findings suggest that EAOC may involve specific molecular mechanisms, which are partially reflected in the sensitivity to chemotherapeutic drugs and must be further investigated.

In this study, the median follow-up time was 60 months. Our results showed that the EAOC patients exhibited significantly longer OS and DFS time than the non-EAOC group (median[IQR], 67[42,101] vs 57[24,88]months and 60[36,89] vs 31[12,69]months, as seen in Tables 3 and 4). Different results have been reported previously. A meta-analysis conducted by Kim et al. showed a better OS rate for EAOC patients (HR 0.778; 95% CI 0.655–0.925) [27]. Orezzoli reported 5-year survival rates of 75% and 34% in two groups of patients with OCCC [28]. These values were in agreement with our results. However, no significant differences in survival rates between two groups of patients were observed by Scarfone [9] and Acién [29]. Multivariate analysis using a Cox regression model revealed that endometriosis remained being the independent impact factor affecting DFS, but not for OS. Besides, FIGO stage, receiving lymphadenectomy, residual disease and chemo-resistance were independent factors affecting the DFS rate in EOC patients. While, FIGO stage, residual disease, the courses of chemotherapy, chemo-resistance and hypertension were independent factors affecting the OS rate. These findings are partially in agreement with the results reported by Garrett et al. [17], Yang et al. [30] and Nasioudis et al. [31]. Collectively, the above findings suggest that the presence of EM might predict a better clinical prognosis for EOC patients. Our data also showed hypertension was the independent predictive factor for OS, which finding had been reported by Minlikeeva et al. [32]. The underlying mechanism of correlation between hypertensions and survival of EOC patients is unclear and need to be explored.

Admittedly, this study did not embrace all variable histotypes of EOC, but only included clear cell and endometrioid subtypes. Garrett et al. analyzed the prognosis of patients with different histological types of OC and observed a better prognosis in patients with endometrioid carcinoma [17]. As we have known, clear cell and endometrioid carcinoma occupied of the massive majority of EAOC, which were also indicated by our previous results. With this in mind, it was reasonable to obtain reliable information about the characteristics for EAOC by analyzing the data extracted from this series of patients.

## Conclusions

This study showed that EOC patients with coexisting EM (EAOC group) were characterized by a younger age of onset, a higher percentage of premenopausal status, lower preoperative CA125 levels, an early tumor stage, a greater extent of satisfying tumor debulking, less opportunity of lymph node metastasis and developing chemo-resistance. The EAOC patients also exhibited better survival, and EM itself was an independent factor affecting DFS. This study was a retrospective case analysis. Certain biases might exist in the results due to inconsistency of the clinical features of the enrolled patients and lack of integrity of the collected clinical data. We expect that further research on this topic will be conducted, particularly at the level of molecular biology to facilitate the screening and early diagnosis of the population at high risk for malignant transformation of EM, ultimately improving the clinical prognosis of the disease.

## Abbreviations

CA-125: cancer antigen 125
DFS: Disease free survival
EAOC: Endometriosis-associated ovarian cancer
EM: Endometriosis
EOC: Epithelial ovarian carcinoma
FIGO: International Federation of Gynecology and Obstetrics
OCCC: Ovarian clear cell carcinoma
OEC: Ovarian endometrioid carcinoma
OS: Overall survival
ROC: Receiver Operating Characteristic
